# Is an image-based eyelid management service safe and effective?

**DOI:** 10.1038/s41433-023-02572-2

**Published:** 2023-05-24

**Authors:** Matthew Gillam, Osman Younus, Shi-Pei Loo, Julie Connolly, Paul Cauchi, Marilena Gregory, Suzy Drummond, Vikas Chadha

**Affiliations:** 1 Chelsea and Westminster Hospitals NHS Foundation Trust, London, UK; 2 NHS Greater Glasgow and Clyde, Glasgow, UK

**Keywords:** Surgery, Medical imaging, Signs and symptoms

## Abstract

**Introduction:**

The COVID-19 pandemic created a requirement for reduced patient contact and reduced capacity in clinics. We previously published results of an Image-Based Eyelid Lesion Management Service (IBELMS) which was found non-inferior to traditional face-to-face clinic at diagnosing lesions and identifying eyelid malignancies. We now present first-year safety and efficacy data from this service.

**Methods:**

Data were collected retrospectively on all patients seen in NHS Greater Glasgow and Clyde eyelid photography clinics from 30^th^ September 2020 to 29^th^ September 2021, including referral source and diagnosis, time to clinic review, treatment and patient outcomes.

**Results:**

808 patients were included in the study. Chalazion was the most common diagnoses recorded (38.4%). There was a statistically significant decrease in mean time from referral to appointment time between the first 4 months and last 4 months of the service (93 days to 22 days, *p* ≤ 0.0001). 266 (33%) of patients were discharged following photographs, 45 (6%) were discharged for non-attendance and 371 (46%) were booked for a minor procedure. 13 biopsy-confirmed malignant lesions were identified; only 3 had been referred as suspected malignancy. 23 patients out of 330 with at least 6 months follow up (7%) were re-referred within 6 months of treatment or discharge; however, none of them with a missed periocular malignancy.

**Discussion:**

Eyelid photography clinics effectively reduce patient waiting times and maximise clinic capacity. They accurately identify eyelid lesions including malignancies with a low re-referral rate. We propose that an image-based service for eyelid lesions is a safe and effective way of managing such patients.

## Introduction

Uptake of tele-medicine has long been a feature in ophthalmic clinical practice; however, the COVID-19 pandemic provided a catalyst to the rapid expansion of virtual services both in ophthalmology and the wider medical world. In March 2020, the NHS was directed to “reduce provision of outpatient services and direct health resources and personnel to acute care [1]” and along with guidance on social distancing, significantly reducing the resources for management of patient with eyelid lesions.

In early 2020, NHS Greater Glasgow and Clyde (NHSGGC) conducted a pilot study comparing usual standard of care (a face-to-face consultation with a consultant ophthalmologist) to a consultation with a hospital optometrist and review of eyelid lesion photographs by a consultant ophthalmologist and a technician led eyelid photograph clinic with images reviewed later by a consultant ophthalmologist [2]. Evaluation of our photography-based service found it to be non-inferior to the traditional consultant-led service, with a low-risk for missed malignancies [2]. Previous studies have also demonstrated the effectiveness of diagnosing eyelid lesions via telemedicine [3, 4].

The department, on the basis of this pilot, decided to implement a technician-led Image Based Eyelid Lesion Service (IBELMS) in September 2020 and we report the data from the first year of this service. Our aims were:

1. To calculate the effect of this service on the waiting time for management of eyelid lesions in NHSGGC
2. To understand the nature of lesions seen within the service and the sources of referral
3. To determine the safety of this service by analysing the numbers of eyelid malignancies that were diagnosed and using a surrogate marker to identify missed malignancies
4. To determine the rate of re-referral following discharge or management within the service

## Methods

This is a retrospective cohort study. All patients vetted to attend the eyelid photography clinic between 30^th^ September 2020 and 29^th^ September 2021 at NHS Greater Glasgow and Clyde were included in the study. Any referrals from other clinicians (such as dermatologists or other ophthalmologists) that explicitly mentioned a ‘suspected malignancy’ were fast-tracked to a separate pathway and hence excluded. Patients who did not consent to undergo clinical photography were also excluded.

The IBELMS clinic pathway started with patients attending for an appointment at the eyelid photography clinic. The trained medical photographers filled in a questionnaire regarding their symptoms, duration of lesion, change in size of lesion and previous history of skin malignancies. The photographer took consent for clinical photography and photographed the lesion and the eyelids. The clinical photographs and completed questionnaires were then reviewed asynchronously by a consultant oculoplastic surgeon. One of the following outcomes were communicated to the patient and referrer by letter: (1) discharge with advice (2) book to a face-to-face clinic (3) book for minor operations clinic (4) book for a procedure in main operating theatre. Written information (for example, hot compresses and massage for chalazia) was included as required.

Data was collected from the board’s electronic medical record (Clinical Portal, Orion Health, Auckland, New Zealand) on the referral source and referrer diagnosis, clinician diagnosis, histological diagnoses (where available) and management. Referral-to-appointment and referral-to-treatment time was also calculated. Re-attendance rates were based upon those patients who had attended the eyelid photography clinic in the first 6 months of the data collection period. Statistical analysis was performed using Microsoft Excel (Microsoft, Seattle, WA, USA) and Medcalc for Windows (Medcalc, Ostend, Belgium). This study formed part of a service evaluation, individual patient consent was therefore not required and the research and ethics committee at NHSGGC did not deem formal approval necessary. The study adheres to the tenets of the Declaration of Helsinki.

## Results

**Fig. 1:**
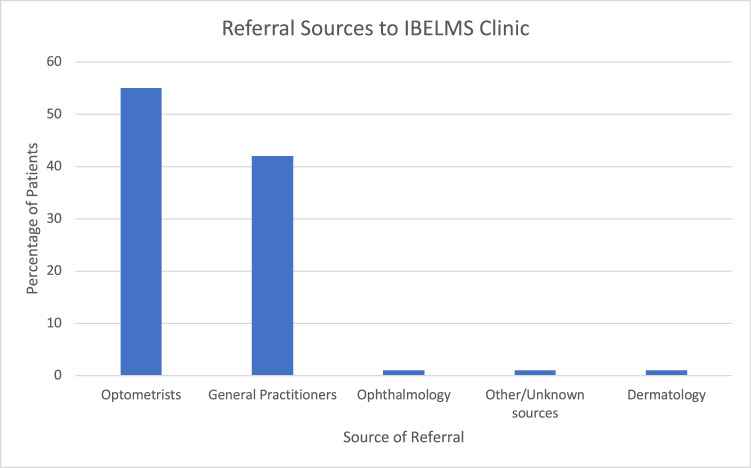
Referral sources. Sources of referral for all patients referred to the IBELMS clinic.

809 patients were included in the study. There was a slight female predominance (54% female, 46% male). Referral sources are shown in Fig. 1. Chalazion (meibomian cyst) was the most common referrer diagnosis (311, 38.4%) with “cyst” of varying types being recorded by the referrer in 121 (15.0%) of cases.

**Fig. 2:**
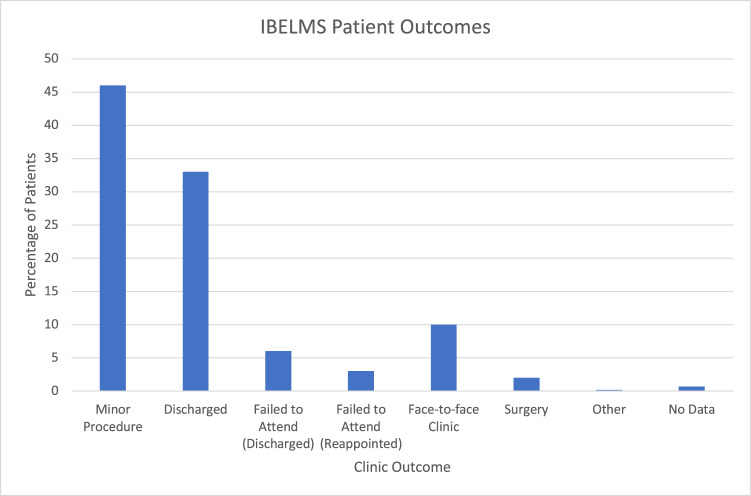
IBELMS Clinic Outcomes. Outcomes from all patients referred to the IBELMS clinic.

808 appointments were offered to 789 patients in the eyelid photography clinic. 45 (5.5%) of appointments were not attended and patients were discharged back to the referrer with the option of re-referral as appropriate. 22 (2.7%) of appointments were not attended but were rebooked and all patients attended a subsequent appointment. Therefore, the non-attendance rate was 8.3% of appointments. 6 (1%) of appointments had insufficient data and 1 patient died prior to attending his appointment following referral. This left 734 patients available for analysis. A breakdown of the outcome of all patients referred to the eyelid lesion clinic is shown in Fig. 2. 32.9% of patients with discharged with advice directly from the clinic and 46.0% were listed directly for an eyelid procedure. A diagnosis from photographs was recorded in 694 cases with chalazion being the most common diagnosis recorded by the consultant ophthalmologist in 290 patients (41.8%). 89.1% of patients did not require a review in a face-to-face clinic before definitive management or discharge was undertaken. If the non-attenders are also included, the service discharged 39.6% of patients and 90.1% did not require a face-to-face clinic appointment before definitive management or discharge.

In the initial few months, the service was catering to the backlog of patients on the waiting list created by the pandemic. During the first 4 months of the service, the average time from referral-to-assessment was 93 days. During the last 4 months of this study period, the mean time from referral to assessment was 22 days (*p* ≤ 0.01, Comparison of Means). The latter is more likely to reflect the waiting time of the service in the long run.

310 (41.8%) patients underwent a surgical procedure. The mean waiting time from referral-to-procedure was 194 days at months 1–4, and 122 days from months 8–12. (*P* ≤ 0.0001, Comparison of Means). Of the patients referred from IBELMS for an eyelid procedure, 22 were found not to require the procedure when they attended giving a false positive rate (patients referred for interventional treatment not requiring it) of 5.7%. However, the lesion (in particular Meibomian cysts) may well have resolved by the time the patient was seen for intervention.

**Fig. 3:**
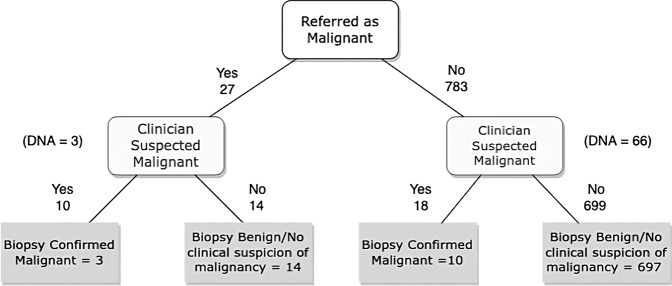
Suspected malignancies. Flowchart for patients with suspected malignancies during IBELMS clinic pathway.

Figure 3 indicates a flowchart of the process for suspicious lesions. In one case, a lesion, classified as a benign seborrheic keratosis after review of photography, returned a histological diagnosis of basal cell carcinoma following excision. In all other cases, there was either a documented clinical diagnosis of malignancy after photo review or the patient was transferred directly into a setting where biopsy could be performed following photograph review. Therefore, we calculate the rate of missed malignancy in the patients who attended as 0.13% (1/808).

Histopathological data was available for 141 patients although 3 of these patients had incomplete clinical data. In 84 (60.9%) of the remaining 138 cases the histological diagnosed matched that recorded by the clinician from the IBELMS clinic. In the remaining 54 cases, the diagnosis differed; however, in 53 of these (98.1%) both the clinical and histopathological diagnoses were benign.

6-month data on re-referral was available for 330 patients. Of these, 24 (7.3%) patients were re-referred. 18 (75%) of re-referrals were for enlarging or non-resolving lesions, 4 (16.7%) for unrelated pathology and 2 (8.3%) at the patient’s request for further advice or discussion. We consider the false negative rate, patients who were initially discharged without face-to-face review or interventional treatment who were later referred and required interventional treatment for their original lesion as 4.5%. No patient was re-referred with suspected malignancy in the periocular region.

## Discussion

This study demonstrates that a photographer-led eyelid lesion clinic is a safe and effective way of assessing patients referred to secondary care with an eyelid lesion which is felt likely to be benign. We demonstrate that such a service can significantly reduce the time patients wait for assessment and interventional treatment if required. We show that it is effective at the identification of eyelid malignancies with a rate of missed malignancy of 0.13%. We consider a 6 months re-referral rate of 9% to be reasonable, especially given around 20% of these re-referrals were for an unrelated pathology. However, our appointment non-attendance rate of 8.3% was slightly higher than the 6.8% recorded in Scotland in the first quarter of 2019/20 [5], the last period for which data is available.

Our figure of 60.9% correlation between clinical and histopathological diagnosis is lower than that reported in series such as Banerjee et al. [6] who reported 90.2% concordance for benign lesions and 67.6% for malignant lesions. This was however based on an in person evaluation of eyelid lesions. Our rate of missed malignancy of 0.13% compares favourably with a study by Izzettinoglu et al. who reported a 2.2% rate of lesions diagnosed as benign clinically which were found to be malignant on histopathology within a series of 408 eyelid lesions [7]. Further none of the 330 patients seen in the first six months of the study period were re-referred with a suspected malignancy. This is also a surrogate marker of the extremely low rate of missed malignancies.

This study was not specifically designed to assess cost implications of our IBELMS service however we feel the service is likely to be more cost-effective than a traditional face-to-face model. Prior to the introduction of the service, a proportion of these patients would have been seen in a face-to-face consultant-led clinic and a proportion would have been referred directly to a nurse-led minor operations clinic. Many patients would have had an image of their eyelid lesion recorded for documentation. In the IBELMS model around 3.5 times as many patients can be reviewed by a consultant in a session than in a face-to-face model (40 IBELMS patients vs 12 patients in a standard clinic profile). The number of patients booked for surgery who do not need or qualify for surgery is likely to be lower with prior secondary care evaluation. These savings are partially offset by the cost of increased medical illustration staff and time in the IBELMS model however. There were no capital costs associated with the IBELMS service as it uses existing equipment and software. We would advocate a study designed to specifically assess cost implications of this model to support introduction in other units.

Several studies have reported on the utility and safety of telemedicine for the assessment of eyelid lesions. Kang et al. [3] in a study of 44 patients showed 91% agreement in diagnosis between in person nurse specialist review and remote consultant review of images and 82% agreement with management. No lesion felt to be malignant on in person review was deemed benign by the remote reviewer. Ah-Kye et al. [8] recently published data from synchronous telemedicine consultations for patients with presumed benign eyelid lesions. In their unit, patients send a self-taken photograph of their eyelid and then receive a video consultation with a clinician to discuss the diagnosis and management. They had a slightly higher rate of patients directly listed for surgery than in our series (57.3% vs 47.9%) however they discharged fewer patients (22.8% vs 33.2%) [8]. They report a very low non-attendance rate at 2.57%. We propose that not having to attend a healthcare facility for any aspect of the consultation is likely to lower non-attendance rates versus our model. However, patient-supplied images and a reliance on the quality of a patient’s device camera versus a medical photographer image may reduce the confidence of a clinician in discharging a patient hence the lower discharge and higher clinic review rate in their series.

We believe this study to be the largest to date looking at asynchronous remote evaluation of eyelid lesions. Whilst it was performed at a single centre, there were a number of photographers and consultant reviewers who evaluated patients so we feel these findings could be replicated at other units and the results are not likely influenced by any individual photographer or clinician. We feel that this study would be representative of the UK population as a whole as it would have captured the majority of referrals with eyelid lesions within the Greater Glasgow area during this period, as no other public sector provider treats patients for periocular lesions in the region. In planning an image-based eyelid lesion service, the reported 7–8% rate of re-referrals must be factored in.

Asynchronous and synchronous telemedicine both have advantages and disadvantages. As demonstrated by Ah-Kye et al.’s study [8] synchronous telemedicine with patients not being required to attend a healthcare facility, non-attendance rates are likely to be lower. However, this model risks excluding the 10% of patients who do not use the internet [9] whereas we feel that our model is accessible to a broader range of patients. It does involve patients attending a healthcare site though so whilst capacity in consultant lead face-to-face clinics is improved, the benefits in sustainability as demonstrated by Ah-Kye et al. [8] would not be replicated with this model. In the future, clinical photographs could be taken in optometry practices which would combine the benefits of a professional standard photograph with a reduction in patient travel. Preliminary data from cloud-based referral platforms suggest the potential to reduce hospital attendance by up to 52% [10]. With synchronous telemedicine, clinicians need to undertake consultations during a set period when appointments are booked whereas with asynchronous telemedicine, clinicians can review records at a time that suits them, facilitating flexible working. Patients do not need to set aside a specific time for an appointment as they do with synchronous telemedicine consultations which many prefer.

Limitations include the fact that the study was retrospective and recording was not complete for all patients. 6 months follow up data was only available for less than half of the studied patients and, given that not all patients had a biopsy or excision procedure performed, the exact diagnosis of their lid lesions cannot be confirmed. The study did not evaluate patient satisfaction rates in this cohort; however surrogate measures for satisfaction, including re-referral rates, were low.

In conclusion, we demonstrate that an image-based asynchronous teleconsultation model for the assessment of patients with presumed benign eyelid lesions is efficient and safe. It Is able to reduce waiting times for an assessment appointment, allow clinicians to work flexibly and minimise use of clinic consultation space whilst accurately identifying periocular malignancies requiring prompt treatment. We recommend that other units with an increasing waiting list of patients with eyelid lesions or restricted by face-to-face clinic capacity consider adopting this model.

## Summary

### What was known before

- Telemedicine is widely used within different subspecialties in ophthalmology Different telemedicine methods have been reported to be successful in the field of oculoplastics Photography-based eyelid lesion clinics are non inferior than face-to-face clinics when diagnosing eyelid lesions.

### What this study adds

- Photography-based eyelid lesion clinics are able to pick up eyelid malignancies reliably Photography-based clinics are an effective way to reduce referral to assessment and referral to treatment times for patients with eyelid lesions Patients assessed with photography are rarely re-referred for further assessment and treatment.

## Data Availability

The data used for the production of this study is available in anonymised form from the authors upon reasonable request.
